# Association of Fucosyltransferase 2 Gene Polymorphisms with Inflammatory Bowel Disease in Patients from Southeast China

**DOI:** 10.1155/2017/4148651

**Published:** 2017-01-12

**Authors:** Hao Wu, Liang Sun, Dao-po Lin, Xiao-xiao Shao, Sheng-long Xia, Ming Lv

**Affiliations:** ^1^Qilu Hospital, Shandong University, Ji'nan, Shandong 250012, China; ^2^Department of Gastroenterology, The Second Affiliated Hospital, Wenzhou Medical University, Wenzhou, China

## Abstract

*Aims*. Fucosyltransferase 2 (*FUT2*) gene potentially affects the constituent of intestinal microbiota, which play a crucial role in the pathogenesis of inflammatory bowel disease (IBD). This study investigated the association of* FUT2* gene polymorphisms with IBD in southeast China.* Methods*. We collected 671 IBD patients and 502 healthy controls.* FUT2* gene polymorphisms (C357T, A385T, and G428A) were determined by SNaPshot. Frequencies of the* FUT2* genotypes, alleles, and haplotype between groups were compared by *χ*^2^ test.* Results*. The allele and genotype frequencies of* FUT2* did not differ between ulcerative colitis patients and controls (all *P* > 0.05). However, mutant allele and genotype of* FUT2* (A385T) were significantly increased in Crohn's disease (CD) patients (*P* = 0.024, OR = 1.271, and 95% CI = 1.031–1.565; *P* < 0.001, OR = 1.927, and 95% CI = 1.353–2.747, resp.). The same conclusion was drawn from* FUT2* (G428A) (*P* = 0.023, OR = 3.324, and 95% CI = 1.108–9.968; *P* = 0.044, OR = 1.116–10.137, and 95% CI = 1.116–10.137, resp.). The haplotype TT formed with “C357T and A385T” was more prevalent in CD patients than in controls (*P* = 0.020, OR = 1.277, and 95% CI = 1.036–1.573). Besides, frequencies of mutant allele and genotype of* FUT2* (A385T) were significantly lower in patients with ileocolonic CD than in those with colonic CD (*P* = 0.001 and 0.002, resp.) and ileal CD (*P* = 0.007 and 0.004, resp.).* Conclusions*.* FUT2* gene polymorphisms and haplotypes were associated with the susceptibility to CD but not UC.

## 1. Introduction

Inflammatory bowel disease (IBD), including ulcerative colitis (UC) and Crohn's disease (CD), has a rising global incidence rate in recent years [1, 2]. Although the pathogenesis of IBD is not yet fully clarified, it has been suggested to involve factors such as the environment, heredity, infection, and immunity. Gene polymorphisms in* NOD2*,* ATG16L1,* and* IRGM* have been commonly accepted as host factors in the predisposition of CD in western countries. However, these gene polymorphisms were not related to the predisposition of CD in Chinese population [3, 4], except for a new variant (P268S) in* NOD2* [5]. Hence, it is of interest to identify new susceptibility gene in IBD in Chinese Han population.

Fucosyltransferase (FUT) 2 gene is located in the q13 region of chromosome 19. The protein encoded by this gene, *α*-(1,2)-fucosyltransferase, is a Golgi stack membrane protein involved in the creation of a precursor of the H antigen, which forms the basis of A and B antigen synthesis. The ABH antigens are the antigens widely expressed in the stomach and small intestine. However, the expression of ABH antigens decreases progressively from the proximal to the distal colon and almost disappears in the rectum in the gastrointestinal (GI) tract [6]. Numerous studies have showed that human histoblood group antigens (HBGA), namely, ABH antigens and Lewis antigens, serve as receptors for norovirus capsid protein attachment and play a critical role in infection [7]. ABH antigens also function as receptors for* Campylobacter jejuni* [8] and rotavirus [9]. Moreover, these antigens can act as a carbon source providing energy for the metabolism of certain bacteria (e.g., the* Escherichia coli*) [10].

It has been suggested that* FUT2* gene polymorphisms may affect the predisposition of celiac disease [11], type 1 diabetes [12], and primary sclerosing cholangitis [13]. McGovern et al. reported in Caucasian population that FUT2 nonsecretor status is associated with Crohn's disease [14]. The role of* FUT2 *gene in UC has also been investigated, but the results seem to lack consistency [11, 15]. Therefore, this study aimed to explore the relationship between* FUT2* gene polymorphism and IBD susceptibility in the Chinese population, which might provide a genetic basis for the mechanism of IBD and find potential new therapeutic targets.

## 2. Materials and Methods

### 2.1. Study Subjects

From March 2012 to March 2014, a total of 671 IBD patients, including 396 UC patients and 275 CD patients, were recruited from The Second Affiliated Hospital of Wenzhou Medical University, The First Affiliated Hospital of Wenzhou Medical University, Wenzhou Central Hospital, and Wenzhou People's Hospital in Wenzhou city, Zhejiang province of Southeast China. The diagnoses of UC and CD were made by means of endoscope, in collaboration with clinical, histopathological, and radiologic findings according to Lennard-Jones Criteria [16]. The severity of UC was evaluated by Truelove and Witt Activity Index [17]. The lesion location and behavior were evaluated on the basis of the Montreal Classification [16]. A total of 502 age- and sex-matched healthy controls were collected at the Health Examination Center of The Second Affiliated Hospital of Wenzhou Medical University, after excluding autoimmune diseases, tumors, and IBD family history. The study protocol was in line with the Treaty of Helsinki and was approved by Ethics Committees of the three hospitals mentioned above. The written informed consent was obtained.

### 2.2. Genomic DNA Extraction and Genotyping Analysis

Approximately 3 mL of the peripheral blood was obtained from each subject into an EDTA tube. DNeasy Blood & Tissue Kit (Qiagen GmbH) was applied for the extraction of genomic DNA from the peripheral blood according to the manufacturer's instructions. Then the genomic DNA was diluted to a concentration of 10 ng/*μ*L and stored at 4°C for subsequent identification of genetic mutations.

The amplification primers of* FUT2* were designed as follows: 5′ TCAACATCAAAGGCACTGGGACC 3′ (forward) and 5′ TGGCGGAGGTGGTGGTAGAA 3′ (reverse). A multiplex SNaPshot assay (Applied Biosystems, California, USA) was employed to determine the genotypes. Firstly, 10 ng of genomic DNA was added to a 10 *μ*L PCR mixture containing 20 *μ*mol dNTPs (Promega, Wisconsin, USA), 0.5 U of FastStart Taq DNA polymerase (Roche, Basel, Switzerland), 1 *μ*L 10x PCR buffer with MgCl_2_ (15 mmol/L) (Roche, Basel, Switzerland), and amplification primers with a terminal concentration of 0.1 *μ*mol/L. The thermal cycler conditions of multiplex PCR amplification were as follows: initial denaturation at 95°C for 5 min and amplification for 35 cycles at 95°C for 30 s, 65°C for 30 s, and 72°C for 1 min, followed by a final elongation step at 72°C for 10 min. Subsequently, the PCR products were examined by electrophoresis in a 2.5% agarose gel. Secondly, we purified the PCR products using a mix of 2 U of Exonuclease I (TaKaRa, Dalian, China) and 1.5 U of shrimp alkaline phosphatase (SAP) (New England Biolabs, Massachusetts, USA) at 37°C for 80 min and then 85°C for 15 min. Thirdly, the multiplex SNaPshot sequencing reactions were performed in a final volume of 7 *μ*L containing 2 *μ*L of purified multiple PCR products, 1 *μ*L of SNaPshot Multiplex Mix, 1 *μ*L of 5x sequencing buffer (Applied Biosystems), and 3 *μ*L of SNaPshot sequencing primers (C357T:** 18T**-TGGCAGAACTACCACCTGAA; A385T:** 38T**-TGGAGGAGGAATACCGCCAC; G428A: CACCGGCTACCCCTGCTCCT). The thermal cycler conditions were an initial denaturation at 96°C for 1 min followed by 25 cycles at 96°C for 10 s, 52°C for 5 s, and 60°C for 30 s. Then the depuration of product was performed with 1 U of SAP at 37°C for 60 min and 75°C for 15 min. Finally, 1.5 *μ*L of SNaPshot products was genotyped in the platform of ABI 3730 Genetic Analyzer before they were mixed with 8 *μ*L of HiDi™ formamide and 0.5 *μ*L of GeneScan-120LIZ size standard (Applied Biosystems). Data were analyzed by GeneMapper 4.0 (Applied Biosystems). In order to guarantee the quality of the study, about 3 percent of the samples were randomly selected and regenotyped by direct sequencing. Consequently, the results from regenotyping and SNaPshot were in complete accordance with the originals.

### 2.3. Statistical Analysis

The data were analyzed by statistical software SPSS 17.0. The* chi-square* test (*χ*^2^) was applied to analyze the accordance with Hardy-Weinberg equilibrium and the differences in the distribution of alleles and genotypes. Software Haploview 4.2 was employed to analyze the linkage disequilibrium and haplotype. A two-tailed *P* value less than 0.05 was considered significant.

## 3. Results

### 3.1. Characteristics of IBD Patients and Controls

The demographic data of UC patients, CD patients, and the controls are presented in Table 1. The 396 UC patients were composed of 250 patients with distal colitis and 146 with extensive colitis in terms of lesion location, while in terms of disease severity they included 225 mild colitis, 117 intermediate colitis, and 54 severe colitis. There were 101 colonic CD, 90 ileocolonic CD, and 84 ileal CD involved in the study. The behavior of CD was composed of 113 nonstricturing, nonpenetrating CD, 71 stricturing CD, and 91 penetrating CD.

### 3.2. Comparison of* FUT2* Gene Polymorphisms between UC Patients, CD Patients, and the Controls

*FUT2* gene polymorphisms of the IBD patients and the controls were explored. The distributions of the genotypes of* FUT2* in UC patients, CD patients, and the controls are in accordance with the law of Hardy-Weinberg equilibrium by *χ*^2^ test (all *P* > 0.05). There were no statistical differences for the allele and genotype frequencies of* FUT2* gene in UC patients compared to the controls (all *P* > 0.05) (Table 2). However, mutant allele and genotype of* FUT2* A385T were significantly increased in CD patients (49.27% versus 43.33%, *P* = 0.024, OR = 1.271, and 95% CI = 1.031–1.565; 27.64% versus 16.53%, *P* < 0.001, OR = 1.927, and 95% CI = 1.353–2.747, resp.) (Table 2). The same conclusion can be drawn from the polymorphism site G428A (1.64% versus 0.50%, *P* = 0.023, OR = 3.324, and 95% CI = 1.108–9.968; 3.27% versus 1.00%, *P* = 0.044, OR = 1.116–10.137, and 95% CI = 1.116–10.137, resp.).

### 3.3. Haplotype Analysis of* FUT2* Gene in IBD Patients and the Controls

**Figure 1:**
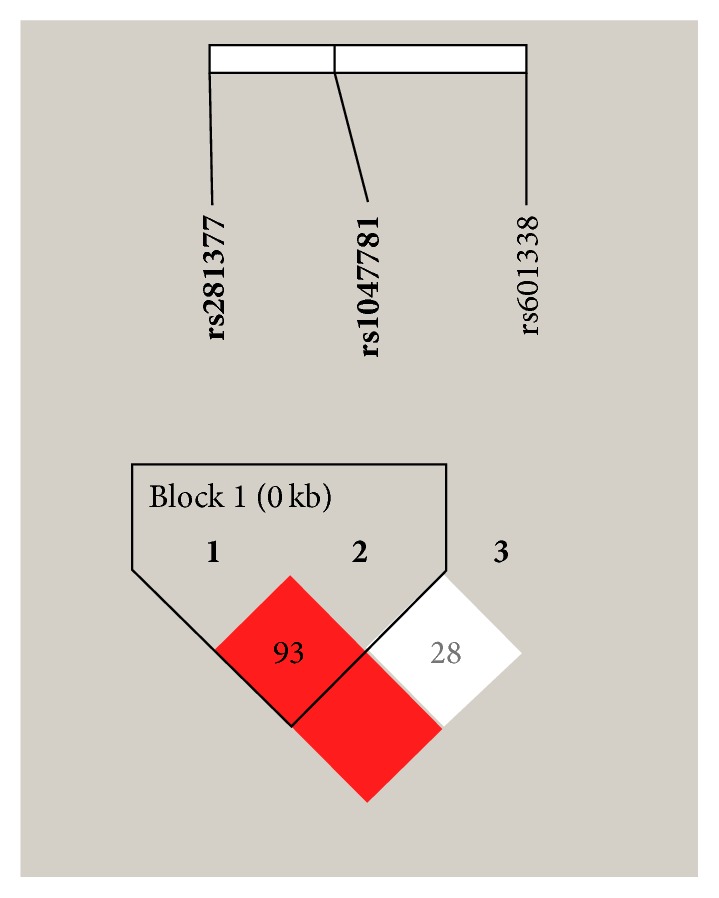
The linkage disequilibrium patterns of* FUT2* gene in Han population from Southeast China. The number in a square represents D′ value for each of the two single-nucleotide polymorphisms (SNPs). Dark color of a square indicates that there is a strong connection between the two SNPs.

In this study, we further applied Haploview 4.2 software for linkage disequilibrium (LD) and haplotype analyses of* FUT2* (C357T, A385T, and G428A). As shown in Table 3, no statistical difference in the frequencies of the haplotypes exists between UC patients and the controls (*P* > 0.05). However, compared to healthy controls, the frequency of haplotype TT, which was formed by C357T and A385T (Figure 1), was significantly increased in patients with CD (*P* = 0.020, OR = 1.277, and 95% CI = 1.036–1.573).

### 3.4. Relationship of* FUT2* Gene Polymorphisms with the Clinical Pathogenic Characteristics of IBD Patients

By stratified analysis, we further explored the association of* FUT2* gene polymorphisms with the clinical features of UC patients and CD patients. In UC patients, the mutant alleles and genotypes of* FUT2* (C357T, A385T, and G428A) did not statistically differ between patients with extensive colitis and distal colitis. Additionally, no significant association was observed between* FUT2* gene polymorphisms and severity of the disease in UC patients (data not shown).

In CD patients, the mutant allele (T) and genotype (AT+TT) of* FUT2* A385T were less prevalent in patients with ileocolonic CD than in colonic CD (41.67% versus 59.41%, *P* = 0.001, OR = 0.488, and 95% CI = 0.324–0.734; 63.33% versus 83.17%, *P* = 0.002, OR = 0.350, and 95% CI = 0.178–0.686, resp.) (Table 4). The frequencies of mutant allele and genotype in* FUT2 *A385T were also lower in patients with ileal CD compared to colonic CD (45.24% versus 59.41%, *P* = 0.007, OR = 0.564, and 95% CI = 0.373–0.854; 64.29% versus 83.17%, *P* = 0.004, OR = 0.364, and 95% CI = 0.183–0.724, resp.) (Table 4). In terms of disease behavior of CD, nevertheless, the frequencies of mutant allele and genotype of* FUT2* polymorphisms showed no difference among stricturing, penetrating and nonstricturing, and nonpenetrating type of CD (data not shown).

## 4. Discussion

As a group of chronic nonspecific intestinal inflammatory disease, IBD is mainly composed of UC and CD. UC is more common in domestic China with a growing incidence rate [1]. The pathogenesis of IBD is not yet clear. Nowadays, most research suggests that the incidence of IBD could be affected by the genetic, environmental, and immune factors or by the intestinal mucosal barrier, intestinal flora, and other factors [18]. The distribution of intestinal bacteria in human is controlled by host genes to some extent. At present,* FUT2* gene is considered to be one of the important genetic factors affecting the intestinal flora [19]. Although one study involving 1503 individuals failed to confirm the association of gut microbiome composition with ABO or secretor status [20], the influence of FUT2 genotype on the gut microbiota has been highlighted not only in healthy individuals but also in patients with Crohn's disease [19, 21, 22].

Expressed mainly in the intestinal tissue, the alpha-(1,2)-fucosyltransferase* FUT2* plays an important role in the formation of the ABH tissue blood group antigen in the intestine. Blood group precursor becomes H antigen after fucosylation by* FUT2* [7]. In the presence of alpha3N-acetylgalactosamine transferase encoded by Gene A, or D-galactosyl transferase encoded by Gene B, H antigen would further become A antigen or B antigen, which together compose the ABH blood group antigen in vivo [7]. Appreciably, ABH blood group antigen is regulated by* FUT2* gene. If ABH blood group antigen is expressed in the saliva, mucous membrane tissue, or secretion of an individual, the phenotype is called ABH secretory type. If not, it is named ABH nonsecretory type. As* FUT2* gene is polymorphic, nonfunctional* FUT2* gene leads to absence of fucosylation activity. The most common* FUT2* nonfunctional locus in the European Caucasian population is G428A [23], while, in the Chinese population, the most common functional site of* FUT2* is A385T [24]. Besides, silent mutation C357T is also commonly found in Chinese nonsecretors [24]. This divergence indicates that the polymorphism of* FUT2 *gene differs among races.

In this study, the distribution of* FUT2* genotypes in the control group was consistent with the Hardy-Weinberg equilibrium law (*P* > 0.05). Mutant allele and genotype of* FUT2* (A385T and G428A) and mutant haplotype TT, formed with* FUT2* (C357T and A385T), were increased in the CD patients compared to the controls, indicating that loss-of-function of* FUT2* gene increased the susceptibility of CD. The results were in line with those from McGovern et al., which suggested that the nonsecretors caused by* FUT2* gene polymorphisms increase of susceptibility of CD [14]. Forni et al. also found that nonsecretor status was associated with CD in Belgian population but not Italian population [25]. Our study also suggested that, compared to patients with colonic CD, frequencies of mutant allele and genotype of* FUT2* A385T were significantly decreased in patients with ileocolonic and ileal CD. Similarly, a study on Japanese population showed that FUT2 secretor status was associated with colonic-type CD and that abnormal expression of blood type antigens was presented only in colonic CD [26]. These findings indicate that the influence of FUT2 gene on CD might be linked to the location of CD. Interestingly, intestinal microbiota are mainly present in the colon and reach their highest biomass in the distal gut [27]. Furthermore, it has been revealed that the expression of Lewis b decreased progressively from proximal to distal colon and disappeared in rectum [6]. We inferred that FUT2 gene might increase the predisposition of colonic CD through its potential influence on the intestinal microbiota.

However, there was no significant difference between the controls and the UC patients in the three* FUT2* mutant alleles or genotype frequencies (all *P* > 0.05). The frequencies of the haplotype between the controls and the UC patients were not significantly different either (*P* > 0.05). Mutant alleles of* FUT2* (C357T, A385T, and G428A) were not significantly different either between the distal colitis group and the extensive colitis group (*P* > 0.05). This is inconsistent with the results from another study, in which the* FUT2* G428A mutant genotype (AA+GA) in the Finland population increased the susceptibility to UC [11]. In addition, studies in China showed that the* FUT2* gene A385T polymorphism is not associated with the susceptibility to UC in the Chinese Han nationality, but the functional mutations of C357T and G428A are in accordance with the susceptibility to IBD of the Han nationality [15]. Thus, it can be inferred that the effect of* FUT2* gene polymorphism on susceptibility to IBD may be closely related to the ethnic differences. In the Chinese Han population,* FUT2*A385T polymorphism is the most common site in the gene, which determines the secretion of ABH antigens.

In conclusion, our study revealed that* FUT2* gene polymorphisms and haplotypes were associated with the susceptibility to CD but not UC.

## Figures and Tables

**Table 1:** Demographic characteristics of IBD patients and the controls.

Characteristics	UC	CD	Controls	*P* value
Total number	396	275	502	
Sex (female/male)	164/232	121/154	226/276	0.549
Age (years) [mean (SD)]	38.98 (16.01)	36.23 (15.25)	37.05 (14.99)	0.051
Age of onset (years) [mean (SD)]	33.04 (13.81)	31.20 (15.67)		0.109
Smoker/nonsmoker	69/327	56/219	82/420	0.376

UC, ulcerative colitis. CD, Crohn's disease. SD, standard deviation. *P* values were calculated by *t-*test or chi-squared test.

**Table 2:** *FUT2* gene polymorphisms in patients with inflammatory bowel disease (IBD) and the controls.

*FUT2*	Controls	UC	CD	UC versus Controls		CD versus Controls	
	*n* (%)	*n* (%)	*n* (%)	OR (95% CI)	*P*	OR (95% CI)	*P*
C357T							
TT	379	292	217	1		1	
TC (additive model)	112	91	57	1.055 (0.759–1.463)	0.742	0.889 (0.608–1.291)	0.521
CC (additive model)	11	13	1	1.534 (0.624–3.839)	0.302	0.159 (0.004–0.996)	0.045
Dominant model (TC+CC versus TT)				1.097 (0.811–1.485)	0.547	0.824 (0.578–1.173)	0.282
Recessive model (CC versus TT+TC)				1.515 (0.671–3.419)	0.314	0.824 (0.578–1.173)	0.282
Allele frequency model (C allele versus T allele)				1.125 (0.861–1.471)	0.387	0.780 (0.563–1.080)	0.134
A385T							
AA	150	122	80	1		1	
AT (additive model)	269	204	119	0.932 (0.691–1.259)	0.648	0.829 (0.586–1.1773)	0.290
TT (additive model)	83	70	76	1.037 (0.697–1.544)	0.858	**1.717 (1.136–2.594)**	**0.010**
Dominant model (AT+TT versus AA)				0.957 (0.719–1.274)	0.764	1.039 (0.752–1.435)	0.818
Recessive model (TT versus AA+AT)				1.084 (0.764–1.537)	0.651	**0.519 (0.364–0.739)**	**0.000**
Allele frequency model (T allele versus A allele)				1.004 (0.832–1.212)	0.964	**1.271 (1.031–1.565)**	**0.024**
G428A							
GG	497	392	266	1		**1**	
GA (additive model)	5	4	9	1.014 (0.271–3.802)	0.983	**3.363 (1.116–10.137)**	**0.044**
AA (additive model)	0	0	0		NA		NA
Dominant model (GA+AA versus GG)				1.014 (0.271–3.802)	0.983	**3.363 (1.116–10.137)**	**0.044**
Recessive model (AA versus GG+GA)					NA		NA
Allele frequency model (A allele versus G allele)				1.014 (0.271–3.789)	1.000	**3.324 (1.108–9.968)**	**0.023**

*P* value, OR, and 95% CI were calculated by chi-squared test. Threshold of *P* value is 0.05. *P* values of statistical significance are in bold. NA, not calculated due to low frequencies. CI, confidence interval; OR, odds ratio. The first row of each comparison group was set as reference.

**Table 3:** Haplotypes of *FUT2* gene in patients with IBD and the controls.

Haplotypes	TA	TT	CA
Patients with UC (%)	42	43	14
Patients with CD (%)	41	49^#^	11
Controls (%)	44	43	13

^#^ *P* value = 0.020, OR = 1.277, and 95% CI = 1.036–1.573.

**Table 4:** Association of *FUT2* gene polymorphisms with the location of CD patients.

FUT2	Colonic	Ileocolonic	Ileal	Ileocolonic versus Colonic		Ileal versus Colonic	
				OR (95% CI)	*P* value	OR (95% CI)	*P* value
C357T							
TT	77	71	69	1		1	
TC (additive model)	23	19	15	0.896 (0.450–1.782)	0.754	0.728 (0.352–1.506)	0.391
CC (additive model)	1	0	0	0.987 (0.963–1.012)	1.000	0.987 (0.963–1.012)	1.000
Dominant model (TC+CC versus TT)				0.859 (0.434–1.699)	0.661	0.697 (0.339–1.436)	0.327
Recessive model (CC versus TT+TC)				0.990 (0.970–1.010)	1.000	0.990 (0.971–1.010)	1.000
Allele frequency model (C allele versus T allele)				0.836 (0.443–1.574)	0.578	0.694 (0.353–1.364)	0.288
A385T							
AA	17	33	30	1		1	
AT (additive model)	48	39	32	**0.419 (0.203–0.861)**	**0.017**	**0.378 (0.179–0.795)**	**0.009**
TT (additive model)	36	18	22	**0.258 (0.114–0.58)**	**0.001**	**0.345 (0.156–0.768)**	**0.008**
Dominant model (AT+TT versus AA)				**0.350 (0.178–0.686)**	**0.002**	**0.364 (0.183–0.724)**	**0.004**
Recessive model (TT versus AA+AT)				**0.451 (0.234–0.871)**	**0.017**	0.641 (0.340–1.208)	0.168
Allele frequency model (T allele versus A allele)				**0.488 (0.324–0.734)**	**0.001**	**0.564 (0.373–0.854)**	**0.007**
G428A							
GG	98	86	82	1		1	
GA (additive model)	3	4	2	1.519 (0.331–6.980)	0.709	0.797 (0.130–4.884)	1.000
AA (additive model)	0	0	0		NA		NA
Dominant model (GA+AA versus GG)				1.519 (0.331–6.980)	0.709	0.797 (0.130–4.884)	1.000
Recessive model (AA versus GG+GA)					NA		NA
Allele frequency model (A allele versus G allele)				1.508 (0.333–6.829)	0.711	0.799 (0.132–4.840)	1.000

Conducted by using unconditional logistic regression analyses, colonic colitis was set as reference compared to ileocolonic colitis and ileal colitis. Variables also included sex, age of onset, and behavior of disease. The first row of each comparison group was set as reference.
